# A Scoping Review of Integrated Blockchain-Cloud (BcC) Architecture for Healthcare: Applications, Challenges and Solutions

**DOI:** 10.3390/s21113753

**Published:** 2021-05-28

**Authors:** Leila Ismail, Huned Materwala, Alain Hennebelle

**Affiliations:** 1 Intelligent Distributed Computing and Systems Research Laboratory, Department of Computer Science and Software Engineering, College of Information Technology, United Arab Emirates University, Al Ain, Abu Dhabi 15551, United Arab Emirates; huned.m@uaeu.ac.ae; 2 National Water and Energy Center, United Arab Emirates University, Al Ain, Abu Dhabi 15551, United Arab Emirates; 3 Independent Researcher, Al Ain, Abu Dhabi 15551, United Arab Emirates; Alain.Hennebelle@gmail.com

**Keywords:** blockchain, cloud computing, electronic health records, health data analytics, healthcare system, security, privacy

## Abstract

Blockchain is a disruptive technology for shaping the next era of a healthcare system striving for efficient and effective patient care. This is thanks to its peer-to-peer, secure, and transparent characteristics. On the other hand, cloud computing made its way into the healthcare system thanks to its elasticity and cost-efficiency nature. However, cloud-based systems fail to provide a secured and private patient-centric cohesive view to multiple healthcare stakeholders. In this situation, blockchain provides solutions to address security and privacy concerns of the cloud because of its decentralization feature combined with data security and privacy, while cloud provides solutions to the blockchain scalability and efficiency challenges. Therefore a novel paradigm of blockchain-cloud integration (BcC) emerges for the domain of healthcare. In this paper, we provide an in-depth analysis of the BcC integration for the healthcare system to give the readers the motivations behind the emergence of this new paradigm, introduce a classification of existing architectures and their applications for better healthcare. We then review the development platforms and services and highlight the research challenges for the integrated BcC architecture, possible solutions, and future research directions. The results of this paper will be useful for the healthcare industry to design and develop a data management system for better patient care.

## 1. Introduction

The healthcare domain has been revolutionized over the last century by technological advancement [1]. This revolution aims to improve the diagnosis of diseases and their causes, quality of medical supplies, medical treatment, and to establish prevention plans on a global scale. The traditional client/server-based healthcare systems [2,3,4,5,6] suffer from security and privacy issues and lead to scattered patient’s medical history delaying patient treatment [7,8]. Moreover, a patient needs to repeat medical tests when moving to another hospital. This increases the cost and time to the patient, and affects the patient’s health due to repeated exposure to tests, such as X-rays and MRIs, that may develop side effects [9]. In addition, healthcare organizations are required to install and maintain infrastructure with up-to-date functionalities while complying with healthcare standards and regulations for the management of Electronic Health Records (EHRs). This leads to a high total cost of ownership. To address, these limitations of the client-server-based approach, the on-premise database migrated to cloud where the health records are maintained by a cloud service provider.

Cloud computing [10] allows convenient and on-demand network access to a shared pool of configurable computing resources. Motivated by the pay-as-use cloud model, medical organizations use cloud computing to manage electronic health records (EHRs), reducing the cost of ownership. The five-year cost of $11 million for an on-premise healthcare system can be reduced to $3.2 million using cloud. This also reduces the infrastructure set-up time from 16-week to 1-week (Healthcare system cost reduction using cloud-based approach: https://ehrintelligence.com/news/how-cloud-ehr-reduces-operating-costs-increases-computing-power, accessed on 27 May 2021). In addition, cloud provides efficient health records’ access to multiple healthcare providers from a shared storage improving patient care. The number of health records is increasing at a rapid pace with the introduction of smart healthcare and IoT with biosensors for personalized patient-centric healthcare. The scalability and elasticity features of cloud computing aid in health records management, which requires powerful computing and large storage, for near real-time patient care. However, a cloud-based system suffers from the issues of security and privacy. Security issue refers to data integrity where the health records are under a constant threat of being modified. Privacy refers to the problem of unobservability, also known as data leakage, in which the patients’ health records are being used without any track [11].

Recent years have witnessed the Blockchain revolution paving the way towards its adoption by many application in the health domain, such as health records management [12,13,14,15,16], medical supply chain management [17,18], and medical insurance claims [19,20]. The characteristics of blockchain make it a great potential for providing a patient-centric healthcare system, involving health stakeholders such as the patients, health professionals, insurance providers, pharmaceutical firms, and health governmental authorities.

From the technical aspect, blockchain is a peer-to-peer distributed system, which enables users to maintain a ledger of transactions that is replicated over multiple user servers [21]. The architecture allows all the network participants, i.e., health stakeholders, to verify and process health data transactions without the need for a trusted third party. In addition, the data stored in the blockchain is immutable, i.e., once the data is stored it cannot be modified or deleted, leading to enhanced security. This immutability enables audit trail, bringing in accountability, adding trust to the system, and alleviating privacy concerns [22,23]. These distinctive features of blockchain have triggered its wide adoption for health records management to address security and privacy issues, while providing access to patient’s health history to multiple stakeholders for patient-centric health services. However, blockchain poses scalability issues as the network grows [24] and consequently more hardware and human resources have to be provisioned for the operation and maintenance of the blockchain platform, thus increasing the health organization’s on-site cost. Moreover, blockchain suffers from the issues of high energy consumption (Bitcoin mining consumes more electricity a year than Ireland: https://www.theguardian.com/technology/2017/nov/27/bitcoin-mining-consumes-electricity-ireland, accessed on 27 May 2021 and Bitcoin energy consumption index: https://digiconomist.net/bitcoin-energy-consumption, accessed on 27 May 2021) adding to blockchain operational cost.

**Figure 1:**
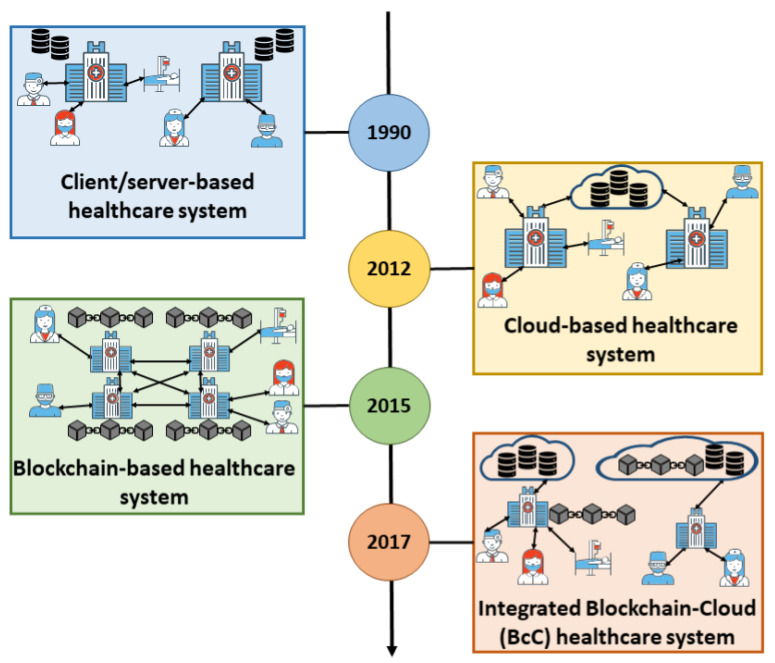
Evolution of healthcare system toward integrated BcC.

Several works in the literature study the strengths and weaknesses of stand-alone cloud or blockchain based healthcare systems. To tackle security and privacy issues prevailing in cloud healthcare systems, the scalability issues inherent from the blockchain algorithms, and to develop more robust solutions for efficient patient care, research efforts have proposed applications using integrated Blockchain-Cloud (BcC) paradigm as shown in Figure 1. However, to the best of our knowledge, there is no work that analyzes the underlying BcC architectures, gives insights, and research directions on how they can be enhanced for a patient-centric healthcare system. The main contributions of this paper are as follows:

- We present the limitations of a healthcare system that is based on either cloud or blockchain and highlight the importance of implementing an integrated BcC system for better patient care.
- We present a scoping review and devise a taxonomy of existing integrated BcC healthcare system architectures into two different types based on the nature of integration. We analyze the effectiveness and limitation of these architectures.
- We compare and analyze Blockchain as a Service (BaaS) platforms provided by different cloud service providers.
- We identify the research challenges prevailing in an integrated BcC healthcare system and possible solutions that are proposed for these issues.

The rest of the paper is organized as follows. Section 2 presents an overview of related work. The concept of cloud computing, blockchain, and the importance of an integrated BcC healthcare system is discussed in Section 3. In Section 4, we present a taxonomy, strengths, and weaknesses of the integrated BcC architectures. Different healthcare applications using the integrated BcC architecture are discussed in Section 5. Section 6 highlights the research challenges in BcC system, and possible solutions. Discussion and conclusion are presented in Section 7 and Section 8 respectively.

## 2. Related Work

The traditional healthcare systems are based on the client/server approach where the patients’ health records are stored in a hospital’s centralized database. Later on, the healthcare system migrated to the cloud-based approach where the patients’ health records are stored and managed in third-party cloud storage by a cloud service provider. This is to solve the issues of scalability, high cost, fragmented patient records, and repeated medical tests existing in the client/server-based approach. Several works in the literature present a review on cloud computing-based healthcare [25,26,27,28]. Hu and Bai [25] in their review classify the work on cloud-based healthcare into three categories depending on the area of focus: (1) framework for data sharing, (2) healthcare applications and (3) security and privacy. The authors highlight the issues of data integrity and confidentiality and suggest that a hybrid cloud model with appropriate access control can be a reliable solution. Ali et al. [26] review applications of the cloud-based healthcare system and highlight the issues of security and privacy with the involvement of a centralized third party, i.e., the cloud service provider. Mehraeen et al. [27] in their review on security challenges in cloud-based healthcare system advise having authentication, authorization, and access control to ensure data security. The authors found that identity management, internet-based access, cybercriminals, authorization, and authentication are the major concerns in the cloud-based healthcare system. Ermakova et al. [28] in their review classify the literature on cloud-based healthcare into 4 contribution categories: (1) framework development, (2) application development, (3) broker development, and (4) security and privacy mechanisms development. However, the cloud-based approach suffers from the issues of centralization, data security, and privacy.

Blockchain, a peer-to-peer network, solves the issues existing in the cloud-based approach. This is because of the immutability, replication, and decentralization characteristics of the blockchain. Work in the literature presents a review of the blockchain-based healthcare system [29,30,31,32,33]. Hölbl et al. [29] analyze the literature based on the contributions, i.e., framework/architecture, algorithm, consensus mechanism, bench-marking metrics, and applicability. The application domains are classified into data sharing, access control, audit trail, and supply chain. The authors highlight the significant use of the technology for data sharing and access control compared to that for supply chain management and audit trail. Kuo et al. [30] present a systematic review of different blockchain platforms with healthcare examples highlighting the platforms’ features such as network type, consensus protocol used, hardware requirement, smart contract support, transaction throughput, scripting language, and open source support. Agbo et al. [31] categorize the work in the literature based on their areas of contributions such as EMRs sharing, supply chain management, biomedical research and education, remote patient monitoring, health insurance claims, and health data analytics. The authors reveal that the application of blockchain technology in the area of healthcare is limited due to the issues of interoperability, scalability, execution time, and patient engagement. Vazirani et al. [32] assess the feasibility of blockchain for efficient EHRs management and concluded that the use of blockchain for healthcare can solve the issues of interoperability, security, and privacy. Hussien et al. [33] categorize the work on blockchain-based healthcare according to their applicability such as clinical/medical data sharing, remote patient monitoring, clinical trials, and health insurance.

Table 1 shows the existing reviews on either cloud-based or blockchain-based healthcare systems. To the best of our knowledge, no work analyzes the integrated BcC architecture for healthcare. In this paper, we highlight the motivation for the integrated BcC architecture for better patient-centric healthcare. In addition, we discuss the evolution of these architectures and capture the assumptions that led to their development. We provide a classification of the integrated architectures and their applications for better healthcare. We then review the BcC developments platforms and services and highlight the research challenges, possible solutions, and future research directions.

## 3. Background and Motivation

In this section, we first explain the fundamental concepts of cloud and blockchain technologies to aid in a better understanding of the rest of the paper. Then, we highlight the motivation of an integrated BcC healthcare system.

### 3.1. Background

#### 3.1.1. Cloud Computing

**Figure 2:**
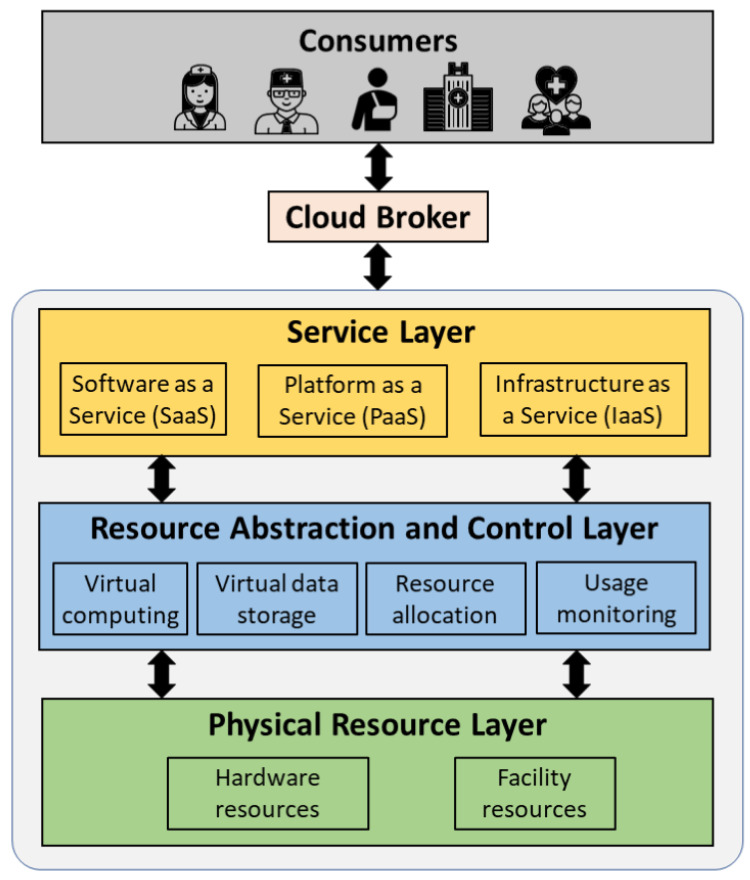
Overview of a cloud system architecture.

Cloud computing technology offers a shared pool of configurable hardware resources and software services over the Internet [10]. These resources can be speedily allocated and released without the system administrator’s intervention. Cloud computing is mainly characterized by on-demand service, rapid elasticity, pay-per-use model, and multi-tenancy. Figure 2 shows the general overview of the cloud system architecture. The architecture consists of (1) cloud consumers that are individual users (patients and allied healthcare professionals) and/or organizations (hospitals) that uses the cloud services, (2) cloud broker that enables the communication between the cloud consumers and the cloud, and (3) cloud entity that makes the cloud services available to the consumers. The cloud consists of three layers: (1) physical resource layer, (2) resource abstraction and control layer, and (3) service layer. The physical layer consists of the hardware resources for processing, storage, and networking, and the facility resources for cooling, ventilation, power, and supply. The resource abstraction and control layer consists of the system components that enable access to the physical resources through a software abstraction. Abstraction components include virtual computing and virtual storage elements. This layer is also responsible for the efficient allocation and usage monitoring of the physical resources. The service layer consists of the interfaces required to access the cloud services. These services by the cloud are classified into Software as a Service (SaaS), Platform as a Service (PaaS), and Infrastructure as a Service (IaaS). SaaS makes software available remotely to multi-tenant users as a web-based service, google mail for example. PaaS provides the environment and tools required to develop web-based applications, Amazon Web Services for example (Amazon Web Services (AWS) - Cloud computing services: https://aws.amazon.com/, accessed on 27 May 2021). IaaS offers virtualized hardware hosted in cloud data centers to the end-users for operations. The hardware involves storage, computing servers, and network components. NTT communications (NTT communications: https://www.ntt.com/en/index.html, accessed on 27 May 2021) is an example of IaaS.

The cloud network can be divided into three main categories:

- *Public cloud:* Allows public access to systems and services without any restrictions and is less secure.
- *Private cloud:* Allows members of the organization that manages the cloud to access the systems and services and is more secure than a public cloud. A private cloud when shared among multiple organizations is known as a community cloud.
- *Hybrid cloud:* Combination of a public and private cloud that enables greater flexibility. The critical and confidential activities can be managed using the private cloud while the general activities can be managed using the public cloud.

With the emergence of cloud computing, the healthcare system migrated from client/ server-based to cloud-based. Cloud solves the issues of fragmented health records and the high total cost of ownership existing in the client/server-based healthcare system. This is thanks to the on-demand access, replication, and pay-as-use characteristics of the cloud. A cloud-based healthcare system is implemented using a private cloud to allow only authorized data access based on access control rights. Several cloud-based healthcare systems are proposed in the literature where a patient/allied health professional can obtain a cohesive view of the patient’s medical history stored in third-party cloud storage [34,35,36]. Although, cloud-based approach improves system scalability and reduces the total cost of ownership, the health records managed by the cloud service provider are under constant security and privacy threats [37,38]. The patients’ records can be easily tampered with or can be accessed without his/her knowledge [11]. Consequently, a more robust healthcare management system is required to address the shortcomings of the cloud-based approach.

#### 3.1.2. Blockchain

Blockchain is a peer-to-peer distributed system that maintains a synchronized ledger of transactions that is replicated over network participants. It was introduced for the exchange of e-currency in a network without the intervention of a third-party [39]. Since then, blockchain has spread in several application domains such as healthcare, education, industry and marketplace, digital media, government, and entertainment. Blockchain has the following properties:

- *Decentralization:* Blockchain eliminates the intervention of a third-party entity for the processing of transactions and maintaining the ledger data. The transactions are validated and executed by the agreement of the majority of the participants that maintain the network.
- *Immutability:* The blockchain is a continuous chain of blocks where a block is connected to its preceding block by including the hash of the latter while hashing the former. A block is composed of a block header consisting of metadata and a block body consisting of valid transactions [21]. If a malicious entity attempts to tamper with the data of a block in past, the hash of the block will change leading to a different hash value than the one used to calculate the hash of the succeeding block. Consequently, the malicious entity needs to re-hash all the subsequent blocks in the chain up till the last block. This re-hashing is compute-intensive especially when there are several replicated copies of the ledger in the network. Thus, any data modification attempt is discouraged leading to immutability.
- *Transparency:* Each operation performed in the network to access the data stored in the ledger is considered as a transaction in the blockchain. Each node in the network that holds the copy of the ledger can track any unauthorized or malicious data access, making the blockchain secure and transparent.
- *Traceability:* The replicated ledger in the blockchain enables efficient tracing of any transaction by the nodes maintaining the ledger. This discourages any malevolent activity, making the network more secure, efficient, and transparent.
- *Consensus:* Each transaction in the blockchain is verified and processed by the agreement of most of the participants holding the ledger copy. This enables transactions between participants who do not know and trust each other.

**Figure 3:**
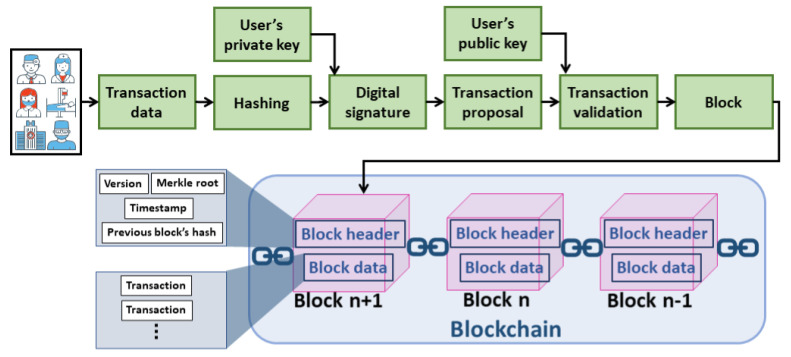
Processing of a transaction in blockchain.

Figure 3 shows how a transaction is processed in the blockchain network. To initiate a new transaction, the transaction data is hashed by the transaction initiator, such as allied health professionals and patients. The digital signature of the transaction is generated by encrypting the hashed data. The encryption is performed using the private key of the transaction initiator. The transaction data and the corresponding digital signature are broadcasted to the network for processing. Each validating node in the network validates the transaction when received. This is by ensuring the authenticity of the transaction initiator and the integrity of the transaction data. The authenticity is verified if the digital signature is successfully decrypted using the transaction initiator’s public key. The integrity is verified if the hashed data obtained from the decryption operation matches the hash of the transaction data. The transaction, if valid, is broadcasted in the network to include it in the block. A miner (node that generates a block) creates a block of the received valid transactions after verifying each transaction for its validity. The selection of a miner that generates a block and the procedure of verifying and appending the generated block to the chain depends on the consensus protocol used by the blockchain network. The consensus protocols in blockchain are classified into compute-intensive-based, capability-based and voting-based [21]. The selected miner generates the hash of the block, also known as the digital signature, and broadcasts the block in the network. The block’s hash is generated by first hashing the block header and then hashing the obtained hashed value. The version in the block header represents the version of the protocol used and the timestamp represents the block generation time. The Merkle root is a single hash value obtained from iterative pair-wise hashing of the transactions in the block data. Each validating node will update their ledger copy by adding the block if valid [21].

The blockchain network can be a public, private, consortium, or hybrid. The public network is the one where any entity can join the network with no prior permission and view the transaction data. On the other hand, a private network, organized by a single organization, is the one where the participation is subjected to prior permission and the data can be accessed based on access control rights. A private blockchain is suitable for healthcare as only authorized members can join the network and the ledger is updated/queried using access control rights. A consortium blockchain is the one where a group of predetermined organizations governs the network. A hybrid blockchain lies between the public and the private ones where the ledger can be viewed by any network participant, but the modifications to the ledger are subject to access control. The distinctive features of the blockchain described above promise a great potential of the technology in the healthcare domain. A blockchain-based healthcare system has the following benefits:

- *Provenance:* The immutable blockchain ledger enables audit trail increasing the trust in the network. Any fraud in the network along with its source can be easily traced. This discourages malicious activities.
- *Protection against natural disasters:* In case of a natural disaster such as forest fires, hurricanes, and floods, a database and its regional replicas might be unavailable. In such a scenario, the globally replicated blockchain ledger can aid in fault tolerance.
- *Real-time data access:* Patient’s health records can be accessed in real-time from the local or the nearest copy of the ledger to avoid life-threatening situations.
- *Accurate patient care:* The cohesive view of a patient’s health records provided by the blockchain enables allied health professionals in better prognosis/diagnosis.

Several blockchain-based healthcare data management systems have been proposed in the literature [12,13,14,15,16]. However, with the increasing amount of health records, the scalability [24,40] and energy consumption (Bitcoin mining consumes more electricity a year than Ireland: https://www.theguardian.com/technology/2017/nov/27/bitcoin-mining-consumes-electricity-ireland, accessed on 27 May 2021 and Bitcoin energy consumption index: https://digiconomist.net/bitcoin-energy-consumption, accessed on 27 May 2021) of blockchain is an issue. In addition, the on-premise blockchain deployment increases the total cost of ownership for healthcare organizations.

### 3.2. Motivation of Integrated BcC for Healthcare

Security and privacy are the main requirements for an effective, trustworthy, patient-centric, and accurate healthcare system. The cloud-based system provides scalability and cost-effectiveness for managing ever-growing health records. However, security and privacy threats become a critical issue due to the involvement of a third-party service provider. Consequently, the healthcare domain seeks a more robust solution for the management of health records. Blockchain, a peer-to-peer network allows transactions between multiple network participants eliminating the need for a third party. Every event in the network is recorded on an immutable ledger, which is replicated over multiple network nodes. Blockchain enables transparent auditing, authorized data access, and immutability, thus providing secure and private management of health records. However, the scalability and the total cost of ownership question the implementation of blockchain in the healthcare domain where the number of health records is continuously increasing. The integrated BcC healthcare system enhances the scalability and reduces the cost while maintaining the security and privacy of the health records.

Recently, there has been growing interest in AI-based healthcare where the health records are analyzed using AI and machine learning algorithms to support allied health professionals with better prognosis and diagnosis of diseases. The accuracy of the AI and machine learning can be improved resulting in a more accurate diagnosis and prognosis of a disease when more instances of data are used for training the models. In this context, an integrated BcC healthcare system would certainly revolutionize the way health professionals provide patient care. The blockchain will facilitate private and secure integration of data from multiple hospitals leading to a rich, secure and accurate database for the AI models and the cloud will enhance the scalability of the system. The incorporation of AI within an integrated BcC healthcare system could lead towards a better patient-centric, secure and private healthcare where the high availability of data from multiple sources, thanks to blockchain, can aid in better diagnosis and prognosis of disease using the AI and machine learning techniques in a scalable cloud environment.

## 4. Taxonomy and Strength/Weaknesses of Integrated BcC Healthcare System Architectures

The individual benefits of cloud and blockchain technologies have led to the emergence of integrated BcC architectures where the limitations of the stand-alone approaches are addressed. In this section, we present an analysis and classification of those architectures. We compare the BcC development platforms and services.

### 4.1. Encapsulated Architecture

**Figure 4:**
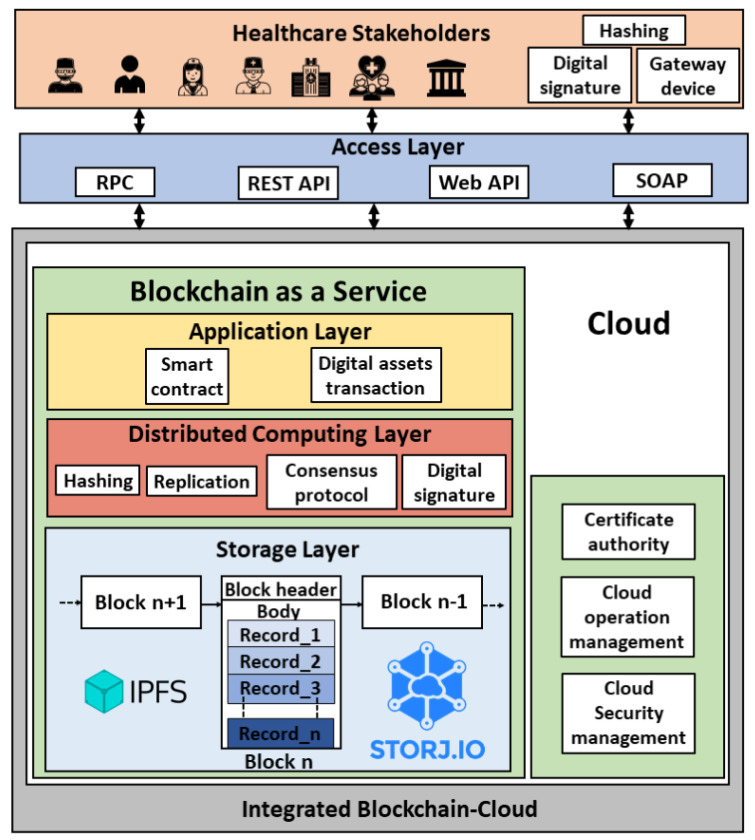
Encapsulated BcC architecture for healthcare.

In this architecture, the blockchain platform and its underlying implementation are encapsulated within a cloud environment as shown in Figure 4. We formulate the encapsulated architecture as stated in Equation (1). This architecture has been proposed by several works in the literature [41,42,43,44,45,46,47]. The network participants (users) are the different health stakeholders such as allied health professionals, patients, health insurance companies, pharmaceutical firms, and the health governmental authorities. The allied health professionals include doctors, nurses, dietitians, medical technologists, therapists, and pathologists. The users can connect to the platform via Remote Procedure Call (RPC), Representational state transfer (REST) Application Programming Interface (API), web API, or Simple Object Access Protocol (SOAP). The health records can be generated by the allied health professional upon patient’s visit or by the patient using sensors. A gateway device is used to process the sensor data. The cloud platform consists of a certificate authority, security management module, and operation management module, in addition to the blockchain as a service. The security management module involves identity and access management, cloud firewall, and web application firewall, and the operation management module includes bill management, data replication and recovery, resource monitoring (CPU, memory, and storage usage) and logs service. The blockchain encapsulated within the cloud consists of an application layer, distributed computing layer, and storage layer. The blockchain ledger in the cloud database is stored using the InterPlanetary File System (IPFS) [48] or storj (Decentralized cloud storage—Storj: https://storj.io/, accessed on 27 May 2021). The health transaction execution flow in this architecture is as follows:

(1) Encapsulatedarchitecture={Cloud∣Blockchain∈Cloud}

**Step** **1:** A transaction initiator (network participant) hashes the health record (transaction payload).
**Step** **2:** The digital signature of the payload is generated by encrypting the hashed transaction.
**Step** **3:** The transaction payload along with the digital signature is broadcasted to the blockchain nodes running in the cloud instances.
**Step** **4:** The transaction is validated, and the block is generated based on the consensus mechanism.
**Step** **5:** The block is updated to the ledger.

Several cloud service providers such as Microsoft Azure (Azure blockchain service: https://docs.microsoft.com/en-us/azure/blockchain/service/overview, accessed on 27 May 2021), Amazon (AWS Blockchain: https://aws.amazon.com/blockchain/, accessed on 27 May 2021), and Oracle (Oracle blockchain platform: https://www.oracle.com/ae/blockchain/, accessed on 27 May 2021) offer cloud-based solutions to help organizations adopt blockchain with ease. In 2015, Microsoft introduced Ethereum Blockchain as a Service (EBaaS) on its cloud platform Azure (Azure’s Ethereum BaaS: https://azure.microsoft.com/en-us/blog/ethereum-blockchain-as-a-service-now-on-azure/, accessed on 27 May 2021). With BaaS, the compute and storage-intensive blockchain runs in the cloud and is managed by the cloud service provider. Blockchain is offered as a service, like any other cloud service, to the consumers (healthcare organizations) to develop and host their blockchain solutions, functions, and smart contracts. The organizations are only charged based on what they use, thanks to the pay-as-use cloud model. For instance, BaaS offered by Amazon Web Services charges $0.067/h for a medium instance peer node, $0.10/GB-month for node storage and data written to the network, and $0.05/GB for more than 150 TB/month data transfer (Amazon managed blockchain pricing: https://aws.amazon.com/managed-blockchain/pricing/, accessed on 27 May 2021). Table 2 shows the encapsulated architecture-based cloud platforms that offer BaaS. It shows the blockchain development platforms supported by these cloud platforms, the type of blockchain network, the consensus mechanism used. In addition, it states whether or not the platform supports the channel. A channel is a private sub-network of communication between specific network participants to perform private and confidential transactions (Channels—Hyperledger Fabric: https://hyperledger-fabric.readthedocs.io/en/release-2.2/channels.html, accessed on 27 May 2021). The channel has its ledger which can only be accessed by the channel members. This is in addition to the main blockchain ledger. The concept of channel is important for healthcare applications in situations such as confidential patient treatment, biomedical research, and formulation of government policies and prevention plans.

In summary, encapsulated BcC healthcare system architecture incorporates blockchain technology and its functionalities within the cloud platform. The healthcare stakeholders have to trust the cloud service provider as the underlying blockchain is implemented and managed by the latter. Consequently, security and privacy issues are not completely addressed by the encapsulated BcC architecture. In this architecture, the system is upgraded by the cloud service provider.

### 4.2. Non-Encapsulated Architecture

**Figure 5:**
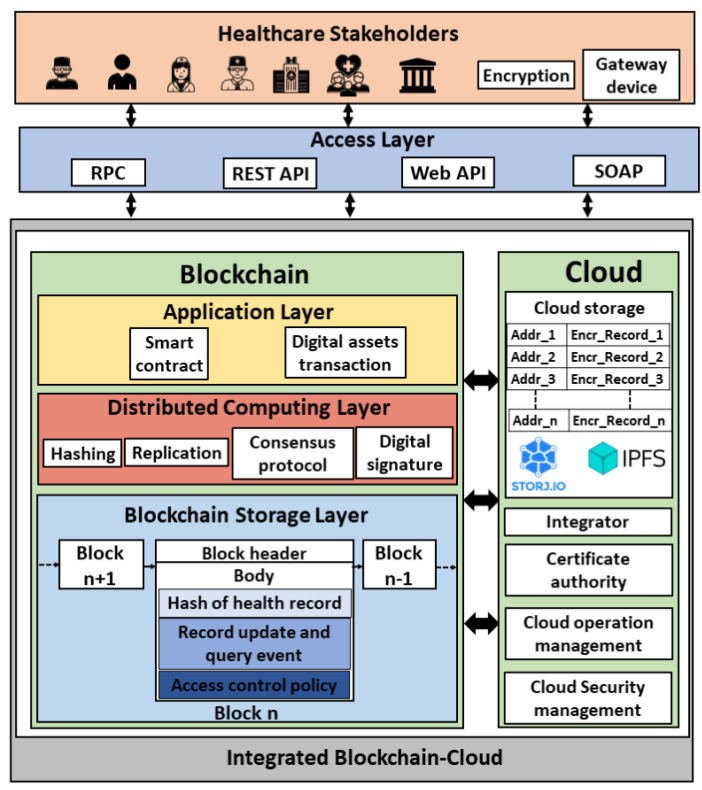
Non-encapsulated BcC architecture for healthcare.

To address the issues of security and privacy existing in encapsulated BcC architecture, non-encapsulated BcC architecture has been proposed in the literature [49,50,51,52,53,54,55,56,57,58,59,60,61,62,63,64,65,66,67,68,69,70,71,72] where the cloud and the blockchain technologies are integrated without encapsulating one into another as shown in Figure 5. We formulate the non-encapsulated architecture as stated in Equation (2). Compared to an encapsulated architecture where the blockchain ledger consisting of health records is managed by the cloud service provider, in non-encapsulated architecture, the health records are managed in the cloud database while the associated meta-data, such as health record’s hash, record update, and query events, and access control policy, is recorded in the blockchain. The medical records in the cloud database are stored using IPFS or storj. The blockchain ledger is replicated across multiple healthcare organizations’ databases. This architecture consists of an additional integrator component compared to the encapsulated one. The integrator enables communication between the cloud and the blockchain platforms. The health transaction execution flow in this architecture is executed as follows:

(2) Non−encapsulatedarchitecture={Cloud∪Blockchain}

**Step** **1:** The health records data is encrypted by the transaction initiator (network participant) and broadcasted to the third-party cloud database.
**Step** **2:** The data is stored in the cloud database.
**Step** **3:** The meta-data of the health record such as the hash of the data, the address in the cloud where the data is stored, and the access control list containing the IDs of the authorized participants is sent to the blockchain by the integrator.
**Step** **4:** The meta-data is recorded in the blockchain as a transaction and the ledge is updated upon consensus.

The off-chain storage for health records in the cloud database enhances the scalability of the system, whereas the meta-data of the transactions in the blockchain ledger aids in security and privacy. The inclusion of the health record’s hash in the blockchain transaction ensures the integrity of the record and the inclusion of record update/query events discourages unobserved access enhancing the system privacy. Table 3 shows the contents of the off-chain storage and the blockchain transactions for the non-encapsulated architecture proposed in the literature. It shows that [49,50,51,52,53,54,55,56,57,58,59,60] store the encrypted health records data in the cloud database, whereas [61] stores the extraction signature along with the encrypted health records data, and [62,63,64,65,66,67,68,69,70,71,72,73] store the clear health records data in the cloud. Extraction signature is the one generated for the health records data after removing the sensitive information from the originally signed data [72]. Regarding the blockchain transaction, some works include the hash of the health records that are stored in the cloud database as transaction payload. This ensures security in terms of data integrity because any modification to the record will result in a new hash value that will be different from the one stored in the blockchain. Other works record either data update and/or query events to the cloud database as transactions in the blockchain. It is crucial to record the cloud data update and query events as blockchain transactions to ensure the privacy of health records because any malicious access to the database will be logged and audit-trailed. Consequently, this discourages malevolent activities. However, very few works [50,59,67,69] consider security and privacy in their non-encapsulated BcC architecture (Table 3). In addition, [53,55,57,61,62,63,64,67,69] include the access control policy in the blockchain transactions for authorized cloud data access.

In summary, non-encapsulated BcC architecture is suitable for healthcare applications as it is more secure and private compared to the encapsulated architecture. The patients’ medical records are stored in the cloud, but the blockchain is implemented outside the cloud and each healthcare stakeholder owns a copy of the ledger that consists of the medical metadata leading to a secure and private healthcare system. In this architecture, the system is upgraded by the cloud service provider hosting the health records. However, in both encapsulated and non-encapsulated architectures, the patients’ medical records are stored in the third-party cloud database which might delay the patient treatment as the data is not locally available to the allied health professionals.

Table 4 shows the strengths and weaknesses of cloud-based, blockchain-based, and integrated BcC healthcare systems. It shows whether or not these systems satisfy the security, privacy, scalability, and real-time data access requirements. The elastic and dynamic characteristics of a cloud-based system offer scalability, but the system suffers from the issues of security, privacy, and real-time data access. A blockchain-based system ensures security, privacy, and real-time data access (from the local copy of the ledger), but is not scalable. The encapsulated BcC system offers scalability as the blockchain is implemented within the cloud. However, cloud storage suffers from security, privacy, and real-time data access issues. The non-encapsulated BcC system is secure, private, and scalable. However, as the health records are stored in the cloud, real-time data access is an issue.

## 5. Healthcare Applications

**Figure 6:**
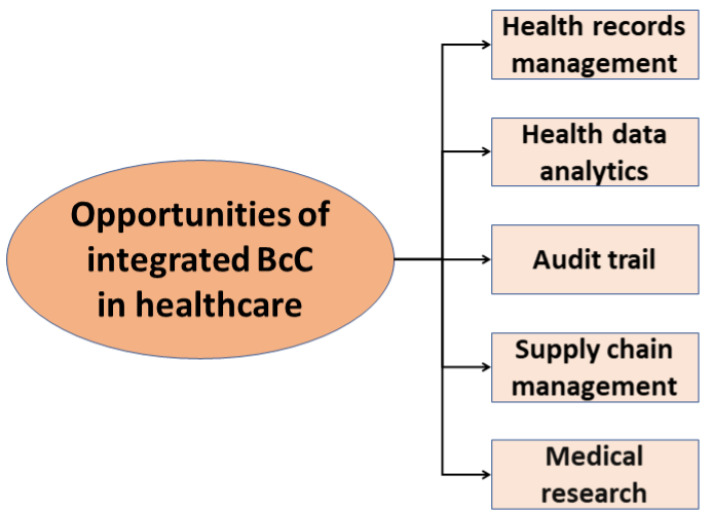
Integrated BcC opportunities in healthcare.

Integrated BcC healthcare system is proposed for different healthcare applications such as health records management [41,42,43,44,45,46,49,50,51,52,53,54,55,56,57,58,59,60,61,62,63,64,65,66,67,68,69,70,71,72], health data analytics [64], audit trail [74], supply chain management [75], and medical research [47] as shown in Figure 6. The healthcare records management systems proposed by [41,42,43,44,45,46] are based on encapsulated BcC architecture, whereas the ones proposed by [49,50,51,52,53,54,55,56,57,58,59,60,61,62,63,64,65,66,67,68,69,70,71,72] are based on non-encapsulated architecture. Nguyen et al. [64] propose a mobile cloud-based Internet of Medical Things framework using blockchain for automated health assessment. The authors use an integrated non-encapsulated BcC system for health data analytics to monitor the progression of neurological disorders. The data stored in the cloud can be accessed through blockchain smart contracts. Zhu et al. [74] proposed a non-encapsulated BcC architecture for the audit trail of healthcare services. The system allows patients to request a paid healthcare service to the cloud. After the end of the service, the patient rates the service provider, and these ratings are stored in the blockchain for audit trail. Celiz et al. [75] propose non-encapsulated healthcare system architecture for supply chain management to track the manufacturing of medical drugs. The data generated using the manufacturing process is stored in the cloud and the data generation events are recorded in the blockchain. Smart contracts are used to ensure the efficient delivery of medical drugs from the source to the destination. For instance, if the shipment is delivered late, then a penalty will be imposed on the supplier using smart contracts. Park et al. [47] proposed an encapsulated BcC architecture for medical research. The authors use blockchain to securely collect and record the evaluation of healthcare remedies such as foods and dietary supplements from different participants. The system uses a cryptocurrency-based reward system to motivate voluntary participation. The proposed system is implemented in the cloud to enhance scalability.

## 6. Integrated BcC Architecture: Research Challenges and Possible Solutions

### 6.1. Scalability

The scalability of the integrated BcC healthcare system architecture is a major issue as the number of health records is continuously increasing. The processing and storage of these records bottleneck the system scalability. Compared to encapsulated architecture, non-encapsulated is more scalable as the health records are stored in the cloud database and only the associated meta-data is recorded as blockchain transactions. However, with the increasing number of participants (healthcare organizations) the scalability of the system is still questionable because of blockchain consensus and replication.

To improve the scalability of blockchain, [76] proposed the concept of sharding network [77]. In this network, the blockchain nodes are divided into shards and the transactions are divided among the shards for parallel verification. Reference [40] proposed a lightweight blockchain architecture for healthcare where the network participants are divided into clusters based on geographical locations. Each cluster has a cluster head and the ledger is only replicated on the cluster heads instead of all network participants. Consequently, the scalability of the system increases. However, none of these solutions has been evaluated and implemented for integrated BcC architecture.

### 6.2. Energy Consumption

The energy consumption of the integrated BcC architecture is a major concern as both cloud computing and blockchain are energy-intensive. The energy consumption of cloud computing is a well-known research area and several works in the literature proposed solutions for energy-efficient cloud computing [78,79]. Regarding blockchain, consensus algorithms consume a significant amount of energy. Several compute-intensive, capability-based, and voting-based consensus algorithms have been proposed for blockchain in the literature [21]. The compute-intensive algorithms such as Proof of Work (PoW) consume a high amount of energy. To address the issue of energy consumption of the compute-intensive algorithms, voting-based algorithms such as Practical Byzantine Fault Tolerance (PBFT) is used. However, PBFT involves a high number of message transfers which might limit the scalability of the system, in particular for the healthcare domain. Moreover, no work examines the communication energy consumption of PBFT due to a high number of message transfers. The high energy consumption of the integrated BcC leads to environmental hazards such as global warming and carbon footprints [80]. Consequently, it is important to have research in this direction.

The possible research directions could be improving the hardware characteristics of the cloud resources to make them more energy-efficient and/or to develop energy-efficient consensus mechanisms such as the cuckoo hash PoW [81]. However, to reduce the environmental impact of these energy-hungry technologies, research should focus on the adaptation of sustainable energy sources such as wind, hydro or solar.

### 6.3. Interoperability

With an increasing trend towards integrated BcC healthcare system architecture, multiple healthcare organizations are using BaaS. To have a cohesive view of a patient’s medical history, all the involved healthcare organizations are thus required to adhere to the same cloud provider. If the healthcare organizations are associated with different cloud service providers, then these service providers must collaborate to use the potential benefits of the integrated BcC architecture. The cloud interoperability standards developed by the National Institute of Standards and Technology (NIST) should be followed by different service providers to support inter-cloud communication [82]. The cloud providers should formalize cooperation agreements based on different levels of interoperability. For instance, the eHealth European Interoperability Framework (eHealth EIF) can be used as a baseline to define and develop legal, organizational, semantic, and technical interoperability levels [83]. However, the eHealth EIF framework should be further expanded for Integrated BcC architecture.

### 6.4. Real-Time Data Access

Real-time patients’ medical records access is crucial for healthcare providers as delay in data access might lead to a patient’s death. In both, encapsulated and non-encapsulated architectures, the medical records are stored in a third-party cloud service provider and there is no local copy of the data with the healthcare professionals. This might lead to a delay in accessing the records depending on the network. Research efforts are required for ensuring real-time data access. One of the solutions can be caching the delay-sensitive health data, such as medication, allergies, results, and treatment plans, in the proximity of healthcare providers for real-time data access in life-threatening situations. The cloud architecture can be expanded by introducing intermediate layer(s) for data storage, such as edge and fog, between the cloud providers and the cloud consumers, i.e., patients, allied health professionals, and health organizations. The inclusion of an edge or fog layer can aid in real-time data access in a cloud-based system [84].

## 7. Discussion

This paper investigated the current research on integrated BcC healthcare system architecture for more efficient and accurate patient care. Cloud technology offers scalability and reduced total cost of ownership, while blockchain technology promises a trust-less decentralized, secure, and private environment. The main objective of this paper is to identify the current state of the art in the integrated BcC architecture and to present the strengths and weaknesses of this architecture.

When implementing an encapsulated or non-encapsulated architecture, the following requirements should be considered:

- *Security:* In the encapsulated architecture the blockchain is encapsulated within the cloud and the underlying blockchain technology is implemented by the cloud service provider. Consequently, the healthcare stakeholders have to trust the cloud service provider for data security as the cloud service provider might tamper with the patients’ records by modifying the underlying blockchain implementation. In this case, an integrated BcC healthcare system is similar to a stand-alone cloud-based healthcare system. On the other hand, in the non-encapsulated architecture, the patients’ records are stored in the cloud database, whereas the blockchain is implemented outside the cloud with each stakeholder having a copy of the ledger. The ledger includes the health records metadata. The stakeholders can track any changes in the health records by the cloud service provider. Therefore, non-encapsulated architecture addresses the issue of data security in healthcare.
- *Privacy:* In the encapsulated architecture, the privacy threat still exists as the cloud service provider might use the patient’s record without the patient’s knowledge. The data query transaction in the blockchain can be removed from the ledger by the cloud service provider as the provider is the one who implements the blockchain and holds the copy of the ledger. On the other, in the non-encapsulated architecture, the privacy of the health records is preserved because the blockchain is implemented outside the cloud and each stakeholder owns a copy of the ledger. Any data query will be recorded in the blockchain ledger, thus making the healthcare system private. Consequently, non-encapsulated architecture addresses the issue of privacy in healthcare.
- *Medical records destruction:* In the encapsulated architecture, the health records are stored in the blockchain ledger and replicated across different network participants. The records stored in the ledger cannot be destructed because of the blockchain’s immutability characteristics. Any attempt to destruct the records will be logged in the ledger. On the other hand, in the non-encapsulated architecture, the health records are stored in the cloud database and not replicated in the ledger. Only the hash of these records and the query/update events are logged in the ledger. Consequently, the destruction of records is possible. The records stored in the cloud database can be destructed and the destruction event will be stored in the ledger.
- *Total cost of ownership:*Table 5 shows that healthcare organizations which implement a non-encapsulated architecture incur the extra cost of recruiting on-site blockchain developer compared to encapsulated architecture.

## 8. Conclusions

In this paper, we highlight the importance of an integrated BcC healthcare system and present a taxonomy of BcC architectures. We also compare the BaaS platform offered by different cloud service providers. In addition, we highlight the issues existing in the integrated architecture and presents possible solutions for future research directions. In summary, the integration of cloud and blockchain for healthcare is promising to cope with the shortcomings of these individual technologies. Further research is still required to enhance the existing architecture to make it more scalable and energy-efficient with inter-cloud communication support.

## Figures and Tables

**Table 1:** Related reviews.

Work	Healthcare System Approach	Area of Focus	Contribution(s)
[25]	Cloud computing	Cloud computing in e-health	Categorization of the cloud-based works, depending on the studied areas, into: (1) framework, (2) application, and (3) security and privacy.
[26]		Opportunities, challenges and applications of cloud-based healthcare	Analysis of cloud computing-based healthcare in terms of opportunities (management and technical), issues (technical, legal, security and privacy), and applications (information processing and monitoring). Discussion of research and implementation implication of the system.
[27]		Security challenges in cloud-based healthcare	Investigation of security challenges and recommendation for secure communication and interoperability in cloud-based healthcare.
[28]		Cloud computing in healthcare	Categorization of the works based on contributions: (1) framework development, (2) application development, (3) broker development, and (4) security and privacy mechanisms development.
[29]	Blockchain	Blockchain in healthcare	Categorization of the works based on contributions (framework/architecture, algorithm, consensus protocol, and bench-marking metric) and applicability (data sharing, access control, audit trail, and supply chain management).
[30]		Blockchain platforms with healthcare as an example	Comparison of blockchain platforms based on the following features: (1) network type, (2) consensus protocol used, (3) hardware requirement, (4) smart contract support, (5) transaction throughput, (6) scripting language, and (7) open source support.
[31]		Blockchain in healthcare	Overview of blockchain and categorization of the work based on contributions: (1) EMRs sharing, (2) supply chain management, (3) biomedical research and education, (4) remote patient monitoring, (5) health insurance claims and (6) health data analytics. Challenges and limitations of blockchain-based healthcare system
[32]		Blockchain implementation in healthcare	Assessment of blockchain feasibility for efficient EHRs management.
[33]		Taxonomy, challenges and recommendations for blockchain-based healthcare system	Overview of blockchain and categorization of the work based on applicability: (1) clinical/medical data sharing, (2) remote patient monitoring, (3) clinical trials, and (4) health insurance. Motivation, challenges, and recommendation for a blockchain-based healthcare system.
This paper	Integrated blockchain-cloud (BcC)	Taxonomy of Integrated BcC healthcare system architectures, challenges and solutions	Importance of integrated BcC healthcare system and taxonomy of existing BcC architectures. Comparison of integrated BcC platforms. Survey of different healthcare applications domains benefited by integrated BcC. Discussion of issues existing in an integrated BcC healthcare system along with possible solutions for future research directions.

**Table 2:** Encapsulated architecture-based development platforms.

Encapsulated BcC Platforms		Blockchain Network	Consensus	Description	Channel Support
Cloud	Blockchain				
Microsoft Azure ${}^{1}$	Ethereum, Hyperledger Fabric, Corda, Chain, and Quorum	Consortium	Istanbul byzantine fault tolerance	Azure Blockchain Service is a BaaS with built-in consortium management that enables quick network deployment and operations with smart contract capabilities. It can be deployed using Azure portal/CLI or through Microsoft Visual Studio Code using the Azure blockchain extension. The services are offered in two tiers: (1) basic, for development and testing, and (2) standard, for deployment.	Yes (Hyperledger Fabric)
Amazon ${}^{2}$	Hyperledger Fabric	Consortium	-	Amazon Managed Blockchain enables easy creation of blockchain networks. The platform uses a voting API, that allows network participants to vote for adding/removing members.	Yes
Oracle ${}^{3}$	Hyperledger Fabric	Hybrid	Raft	Oracle Blockchain Platform enables blockchain configuration, development and execution of smart contracts, and monitoring through a web console. External applications update/query via client SDKs or REST API calls.	Yes
IBM ${}^{4}$	Hyperledger Fabric	Private, public and hybrid	Pluggable consensus	IBM Blockchain Platform allows to develop, test and deploy blockchain applications with smart contract capabilities using Visual Studio code extension. The platform supports multiple languages for the development of smart contracts.	Yes
Google ${}^{5}$	Ethereum	Hybrid	Configurable consensus	Google blockchain enables deployment of blockchain applications with easy API integration. It allows the use of a traditional SQL database for blockchain data update/query.	No
SAP ${}^{6}$	Multichain, Hyperledger Fabric and Quorum	-	-	SAP Cloud Platform Blockchain Service enables development and deployment of blockchain applications from scratch, allows to link external blockchain nodes to the cloud or to connect an external blockchain to SAP’s powerful memory data platform, HANA.	Yes (Hyperledger Fabric)
Hewlett- Packard (HP) ${}^{7}$	Ethereum	-	-	HPE Mission Critical Blockchain enables fault tolerant and highly scalable blockchain applications development with smart contract integration.	No
Alibaba ${}^{8}$	Hyperledger Fabric, Ant and Quorum	Consortium	-	Alibaba Cloud BaaS is developed on top of Alibaba cloud container service for Kubernetes clusters enabling quick development and deployment of blockchain solutions. Alibaba Cloud BaaS API allows users to manage the blockchain objects and cloud resources.	Yes (Hyperledger Fabric)
Huawei ${}^{9}$	Hyperledger Fabric	Consortium	Solo, fast byzantine fault tolerance, and Kafka	Huawei Blockchain Service based on Huawei containers enables easy creation, deployment, and management of blockchain solutions.	Yes (Hyperledger Fabric)
Baidu ${}^{10}$	Permissioned Ethereum, Hyperledger Fabric, and Baidu XuperChain	-	Pluggable consensus	Baidu BaaS enables easy development and deployment of blockchain applications with multichain and smart contracts features.	Yes

^1^ Azure blockchain services https://docs.microsoft.com/en-us/azure/blockchain/service/overview, accessed on 27 May 2021; ^2^ AWS Blockchain https://aws.amazon.com/blockchain/, accessed on 27 May 2021; ^3^ Oracle blockchain platform https://www.oracle.com/ae/blockchain/, accessed on 27 May 2021; ^4^ IBM blockchain https://www.ibm.com/ae-en/blockchain, accessed on 27 May 2021; ^5^ Google cloud BaaS https://cloud.google.com/blog/products/data-analytics/building-hybrid-blockchain-cloud-applications-with-ethereum-andgoogle-cloud, accessed on 27 May 2021; ^6^ SAP blockchain applications and services https://www.sap.com/mena/products/intelligenttechnologies/blockchain.html, accessed on 27 May 2021; ^7^ HP blockchain solutions https://www.hpe.com/us/en/solutions/blockchain.html, accessed on 27 May 2021; ^8^ Alibaba cloud blockchain https://www.alibabacloud.com/products/baas, accessed on 27 May 2021; ^9^ Huawei blockchain service https://www.huaweicloud.com/intl/en-us/product/bcs.html, accessed on 27 May 2021; ^10^ Baidu blockchain service https://github.com/xuperchain/xuperchain, accessed on 27 May 2021.

**Table 3:** Contents of off-chain cloud storage and blockchain transaction in non-encapsulated BcC architectures proposed in the literature.

Work	Cloud Database	Blockchain Transaction			
		Transaction Types		Inclusion of Health Record’s Hash	Access Control Policy
		Record Update Event	Record Query Event		
[49]	Encrypted health record	✓	✓	✗	✗
[50]		✓	✓	✓	✗
[51]		✓	✓	✗	✗
[52]		✓	✗	✓	✓
[53]		✓	✗	✗	✗
[54]		✗	✗	✗	✗
[55]		✓	✗	✓	✓
[56]		✓	✗	✓	✗
[57]		✓	✗	✗	✗
[58]		✓	✗	✓	✓
[59]		✓	✓	✓	✗
[60]		✓	✓	✗	✗
[61]	Encrypted health record and the extraction signature	✓	✓	✗	✓
[62]	Health record	✓	✓	✗	✓
[63]		✓	✗	✓	✗
[64]		✗	✓	✗	✓
[65]		✓	✗	✗	✗
[66]		✓	✗	✓	✗
[67]		✓	✓	✓	✓
[68]		✓	✓	✗	✗
[69]		✓	✓	✓	✓
[70]		✓	✓	✗	✗
[71]		✗	✓	✗	✗
[72]		✓	✓	✗	✗

**Table 4:** Strengths and weaknesses of cloud-based, blockchain-based and integrated BcC healthcare systems.

Healthcare System		Security	Privacy	Scalability	Real-Time Data Access	Remarks
Cloud-based		✗	✗	✓	✗	The system scales but suffers from security and privacy issues. The health records can not be accessed in real-time as they are stored in the cloud.
Blockchain-based		✓	✓	✗	✓	The system ensures security and privacy, and enables real-time of the health records from the local copy of the ledger. However, it does not scale.
Integrated BcC	Encapsulated	✗	✗	✓	✗	The system scales but suffers from security and privacy issues. The health records can not be accessed in real-time as they are stored in the cloud.
	Non-encapsulated	✓	✓	✓	✗	The system scales and ensures security and privacy. The health records can not be accessed in real-time as they are stored in the cloud.

Security: ✓→ the system ensures data integrity and ✗→ the system does not ensure data integrity. Privacy: ✓→ the system ensures data privacy and ✗→ the system does not ensure data privacy. Scalability: ✓→ the system scales when the number of nodes increases and ✗→ the system does not scale. Real-time data access: ✓→ the system allows real-time access of health records and ✗→ the system does not allow real-time access of health records.

**Table 5:** Cost for encapsulated and non-encapsulated BcC architectures.

BcC Architecture	Cost	
Encapsulated	Node	$2785.68 */year
	Transaction	$0.0001 * (50 transactions/day are not charged)
	Cloud storage (ledger)	$0.6 */GB/year
Non-encapsulated	Node	≈$1000 ** (4 years maintenance)
	Cloud storage (health records)	$0.00972 ***/GB/year
	Blockchain developer	$136,000/year [85]
	Operation (energy)	$8309.7 ****/year

^*^ Prices are based on Microsoft Azure Blockchain as a Service for U.S. East deployment [86]. ^**^ Price is for Microsoft surface laptop (8GB RAM and 128GB SSD) [87] based on Bitcoin node requirement [88]. ^***^ Price is based on Microsoft Azure data lake storage [89]. ^****^ The price is based on annual energy consumption of 63 terawatt-hours by a Bitcoin node [90,91]. The average electricity rate of 13.19 cents per kilowatt hour (kWh) in the United States is considered [92].
